# The *Arabidopsis* SAL1-PAP Pathway: A Case Study for Integrating Chloroplast Retrograde, Light and Hormonal Signaling in Modulating Plant Growth and Development?

**DOI:** 10.3389/fpls.2018.01171

**Published:** 2018-08-08

**Authors:** Su Y. Phua, Dawei Yan, Kai X. Chan, Gonzalo M. Estavillo, Eiji Nambara, Barry J. Pogson

**Affiliations:** ^1^ARC Centre of Excellence in Plant Energy Biology, Research School of Biology, The Australian National University, Canberra, ACT, Australia; ^2^VIB Center for Plant Systems Biology, Ghent, Belgium; ^3^Department of Cell & Systems Biology, University of Toronto, Toronto, ON, Canada; ^4^CSIRO Agriculture and Food, Black Mountain, Canberra, ACT, Australia

**Keywords:** phosphoadenosines, chloroplast retrograde, light, hormones, signaling, plant growth

## Abstract

Plant growth and development are dependent on chloroplast development and function. Constitutive high level accumulation of a chloroplast stress signal, 3′-phosphoadenosine-5′-phosphate (PAP), confers drought tolerance to plants, but slow downs and alters plant growth and development. PAP, a by-product of sulfur metabolism, is maintained at very low levels by the SAL1 phosphatase during vegetative growth of *Arabidopsis* and accumulates in rosettes during drought and excess light. Eight independent forward genetic screens in *Arabidopsis* identified SAL1 as the regulator of multiple phenotypes related to stress responses, hormonal signaling and/or perception. In this perspective article, we collate all the *sal1* phenotypes published in the past two decades, and distill the different pathways affected. Our meta-analysis of publicly available *sal1* microarray data coupled to preliminary hormonal treatment and profiling results on *sal1* indicate that homeostasis and responses to multiple hormones in *sal1* are altered during rosette growth, suggesting a potential connection between SAL1-PAP stress retrograde pathway and hormonal signaling. We propose the SAL1-PAP pathway as a case study for integrating chloroplast retrograde signaling, light signaling and hormonal signaling in plant growth and morphogenesis.

## Introduction

Plants are autotrophs that capture light energy from the sun via photosynthesis. The quantity (intensity) and quality (wavelength) of light can affect the assembly and efficiency of photosynthetic machineries in the chloroplasts (Dietz, 2015), which in turn will determine the amount of carbon available for plants to convert into energy for growth and survival. When optimizing and fine-tuning resource allocation for photosynthesis, plants have to account for both the efficiency of light-harvesting through to electron transport chain under limiting conditions such as low light intensities, and the risk of photodamage by reactive oxygen species (ROS) when conditions flip to the other extremes of excessive light exposure and drought. As such, it has been proposed that the chloroplast can act as an environmental sensor that subsequently triggers downstream mechanisms for adjustment of plant function and growth according to the environment. Hence, constant communication in the form of anterograde (nucleus to organelles) and retrograde (organelles to nucleus) signaling takes place between chloroplasts and the nucleus to coordinate and maintain cellular function (Chan et al., 2016b).

Leaf/rosette growth and development appears closely associated with chloroplast development and signaling, as well as with the quantity and quality of light and hormonal levels. Phototropism is a classic example of this, which requires mature chloroplasts (Jin et al., 2001), light signaling and hormonal regulation to take place (Fankhauser and Christie, 2015). There is emerging evidence for developmental abnormalities resulting from changes in chloroplast development (Larkin, 2014; Pogson et al., 2015) and from the presence of cells with non-detectable plastids (Chen et al., 2009). Chloroplasts derived from undeveloped proplastids can propagate via division in a light-responsive manner (Hashimoto and Possingham, 1989; Pogson et al., 2015). Both chloroplast development and plant growth are light-dependent, involving phytochrome-mediated signaling (Okello et al., 2016); and all of these are respectively linked to growth hormonal signaling (Halliday and Fankhauser, 2003; Lau and Deng, 2010; Jiang et al., 2012; Yoshizawa et al., 2014). The key growth-regulating hormones in plants are gibberellic acids (GA), brassinosteroids (BR), and auxin; and some of this hormonal signaling pathway such as GA- and BR-signaling has been demonstrated to crosstalk with light signaling pathway in modulating plant growth (de Lucas et al., 2008; Feng et al., 2008; Bai et al., 2012). However, direct connections that integrate chloroplast retrograde signaling, light signaling and hormonal signaling in regulating plant growth and development are yet to be defined.

Altered levels of chloroplast retrograde signal(s), functioning in either chloroplast biogenesis or operation, ultimately contribute to changes in plant growth and development. For example, *Arabidopsis* mutants over-accumulating plastid signals such as heme (Chory et al., 1989; Woodson et al., 2011; Espinas et al., 2012), methylerythritol cyclodiphosphate (MEcPP) (Gil et al., 2005; Xiao et al., 2012; Bjornson et al., 2017), dihydroxyacetone phosphate (DHAP) (Chen and Thelen, 2010; Vogel et al., 2014), or 3′-phosphoadenosine 5′-phosphate (PAP) (Rossel et al., 2006; Wilson et al., 2009; Estavillo et al., 2011) are smaller in rosette size with varying extents of altered rosette morphology. As some of these retrograde signals such as MEcPP (Xiao et al., 2012) and PAP (Estavillo et al., 2011) accumulate upon excess light exposure, perhaps these chloroplast retrograde signaling mutants could be used to dissect if and how chloroplast retrograde, light and hormonal signaling crosstalk with one another to impact on plant morphogenesis.

PAP in *Arabidopsis* is generated from the secondary sulfur metabolism pathway (Kopriva and Gigolashvili, 2016) when sulfotransferases (SOTs) transfer the sulfate group from 3′-phosphoadenosine-5′-phosphosulfate (PAPS) to other acceptor molecules such as desulfoglucosinolates, salicylic acid (SA), and BR (Chan et al., 2013). Under standard growth conditions, PAP is maintained at very low levels by the SAL1 phosphatase, which degrades PAP into adenosine monophosphate (AMP) and inorganic phosphate (Pi). This degradation occurs in chloroplasts and mitochondria, where SAL1 is localized (Chen et al., 2011; Estavillo et al., 2011). Redox-regulation of SAL1 activity allows for its inactivation during oxidative stress in these organelles, causing accumulation of PAP under drought and excess light stresses (Estavillo et al., 2011; Chan et al., 2016a). Genetic studies demonstrate that PAP is able to move intracellularly, targeting nuclear exoribonucleases (XRNs) to up-regulate stress-responsive genes during drought (Estavillo et al., 2011). Multiple independent studies on PAP-accumulating *sal1* mutants suggest additional roles for this metabolite and its catabolic enzyme.

## Loss-Of-Function Mutations in *Arabidopsis sal1* Give Rise to Diverse Phenotypes

Altered phenotypes related to stress (Lee et al., 1999; Xiong et al., 2001, 2004; Wilson et al., 2009), RNA metabolism (Gy et al., 2007), nutrient uptake (Hirsch et al., 2011), leaf morphology (Robles et al., 2010) or plant hormones (Rodríguez et al., 2010; Zhang et al., 2011) have been associated with mutations in *SAL1*, also known by a diversity of gene names arising from the different alleles characterized (**Supplementary Table S1**) – *hos2* (*high expression of osmotically responsive genes*), *fry1* (*fiery 1*), *alx8 (altered expression of APX2 8)*, *ron1* (*rotunda 1*), *fou8* (*fatty acid oxygenation up-regulated*), and *supo1* (*suppressor of PIN1 overexpression phenotype*). Despite the different ecotype backgrounds such as Col-0 (*fry1-4*, -5, *-6*, *alx8*, *fou8*, *supo1*) (Rossel et al., 2006; Gy et al., 2007; Wilson et al., 2009; Rodríguez et al., 2010; Zhang et al., 2011), L*er* (*ron1*) (Robles et al., 2010), C24 (*hos2*, *fry1-1. -2, -3*) (Lee et al., 1999; Xiong et al., 2001, 2004), and Ws (*fry1-7*) (Hirsch et al., 2011), all *sal1* knockout mutants consistently display the following developmental phenotypes: delayed development and flowering, more compact rosette with shorter petiole length and rounder leaf shape. Additionally, *sal1* was observed to have thicker leaves (Wilson et al., 2009), open venation patterning on leaves and compromised apical dominance based on its increased number of secondary inflorescences compared to wild type (Robles et al., 2010). Altered root morphology was also observed in *sal1* mutants (Robles et al., 2010; Hirsch et al., 2011; Zhang et al., 2011). These growth defects coincide with *SAL1* expression being detected in all cell types of wild type *Arabidopsis* throughout development, with higher expression in vascular or vein tissue of leaves and stamens of flowers (Xiong et al., 2001).

Some *sal1* growth phenotypes are related to light responses. Kim and von Arnim (2009) showed that *sal1* hypocotyl elongation responded similarly to wild type under white light, but was hypersensitive to low-intensity red light, and to a lesser extent, far red and blue light respectively. Crossing a light perception mutant, *phytochrome b* (*phyb*), which has much longer petioles, with the *sal1* mutant yielded a partial reversion of the altered *sal1* rosette morphology. Additionally, Chen and Xiong (2011) demonstrated that an additional *long hypocotyl 5* (*hy5*) mutation in the *sal1* mutant can suppress the enhanced light sensitivity of *sal1* hypocotyl elongation, but not its altered rosette phenotype. This suggests that *sal1* may indeed exhibit altered light perception, signaling or response. As circadian regulation is closely regulated by light signaling (Millar, 2004; Dalchau et al., 2010; Bordage et al., 2016), the effect of PAP accumulation on slightly delaying circadian period (Litthauer et al., 2018) provides further evidence for potential crosstalk between SAL1/PAP and light signaling, although the mechanism remains unclear. Interestingly, a decrease in transitory starch granules was detected in *sal1* chloroplasts (Wilson et al., 2009); whether this could be due to this recently reported circadian phenotype of *sal1* deserves further investigation since starch metabolism is also closely associated to light and circadian regulation (Webb and Satake, 2015).

The discovery of PAP as a chloroplast stress retrograde signal was initiated when a *sal1* knockout mutant *alx8* was identified as having constitutive up-regulated expression of *ascorbate peroxidase 2* (*APX2*) – a chloroplast stress marker gene (Rossel et al., 2006; Wilson et al., 2009; Estavillo et al., 2011). Soil-grown *sal1* plants can survive 50% longer than wild type during a terminal drought by retaining water more efficiently and maintaining photosynthetic activity (Rossel et al., 2006; Wilson et al., 2009). Consistent with its improved drought tolerance, *sal1* has lower stomatal conductance (Rossel et al., 2006; Pornsiriwong et al., 2017), higher accumulation of various osmoprotectants such as putrescine (Wilson et al., 2009), antioxidants and ROS detoxifying enzymes (Rodríguez et al., 2010), jasmonic acid (JA) (Rodríguez et al., 2010) and an inconsistent increase in abscisic acid (ABA) (Rossel et al., 2006; Pornsiriwong et al., 2017). The stress- and ABA-related phenotypes of *sal1* were initially hypothesized to be due to inositol polyphosphates (Xiong et al., 2001), which are candidate secondary substrates of SAL1 *in vitro* (Quintero et al., 1996). This initial hypothesis has been discounted by multiple independent studies that have shown the primary *in vivo* substrate of SAL1 is PAP, with any change in inositols likely to be indirect secondary effects of SAL1 inactivation (Kim and von Arnim, 2009; Rodríguez et al., 2010; Chen et al., 2011; Estavillo et al., 2011; Lee et al., 2012; Litthauer et al., 2018).

The SAL1-PAP retrograde pathway has been shown to interact with the ABA signaling pathway. Pornsiriwong et al. (2017) demonstrated that PAP-XRN signaling restores ABA-responsiveness of ABA-insensitive mutants [*ABA insensitive 1* (*abi1*), *open stomata 1* (*ost1*)] for stomatal closure; and genetic and exogenous manipulation of PAP increase ABA-responsiveness of seed germination in *Arabidopsis*. Meanwhile, Chen et al. (2011) observed that the up-regulated ABA signaling in *sal1* requires functional ABA Hypersensitive 1 (ABH1), an mRNA cap binding protein that is involved in ABA signaling. These are in line with Wawer et al. (2018)’s observation that both mRNA decapping and 5′–3′ decay contribute to the regulation of ABA signaling. It will be interesting to further dissect the details of how SAL1-PAP intersects with ABA signaling pathway *via* its impact on RNA metabolism and whether or not other RNA-independent signaling components are involved.

## Complex Interactions Between Sal1-Pap, Plant Hormones and Growth

Evidence presented thus far indicate that the SAL1-PAP chloroplast retrograde pathway crosstalks with light signaling for growth regulation and ABA signaling for stress response regulation. There are other biochemical and physiological phenotypes of *sal1*: earlier onset of increase in JA levels during vegetative growth (Rodríguez et al., 2010), altered auxin perception/response (Robles et al., 2010), reduced sensitivity toward ethylene (Chen and Xiong, 2010), altered sulfur metabolism (Lee et al., 2012) and enhanced susceptibility toward pathogen attacks (Bruggeman et al., 2016; Ishiga et al., 2017). Evidently, multiple hormonal homeostasis and/or signaling are altered in *sal1* and it is common for hormones to crosstalk with one another in coordinating plant growth and stress responses (Jaillais and Chory, 2010; Depuydt and Hardtke, 2011). Therefore, we hypothesized that SAL1-PAP retrograde pathway could also interact with growth hormonal signaling pathway for modulating plant growth.

**FIGURE 1:**
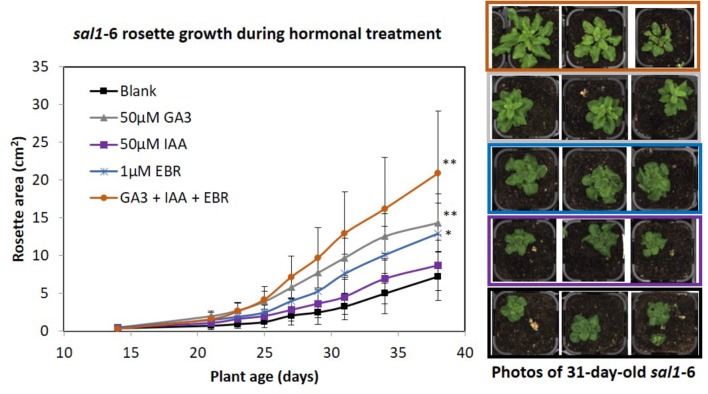
Rosette growth of *sal1* mutant under different hormonal treatment. Soil grown *sal1-6 (fry1-6* allele) mutant were sprayed with different hormones (50 μM of GA_3_, 50 μM of IAA, or 1 μM of EBR individually, or all of the three hormones together) or blank control [containing 0.5% (v/v) ethanol, which was used to dissolve the commercially available hormones, and 0.1% (v/v) Tween-20] from 2 weeks old onwards (three times per week). The rosette growth since treatment were quantified via LemnaTec Scanalyzer for 3.5 weeks (until bolting stage) based on rosette area (full method see, Phua et al., 2018). Average rosette area ± SD of at least 10 plants per treatment are shown. Significant differences from blank treatment at 38 days old (ANOVA – Dunnett test [GraphPad InStat]) are indicated by ^∗∗^*p* < 0.01 and ^∗^*p* < 0.05. Example photos of *sal1* plants at 31 days old (after 16 days of hormonal treatment).

We investigated whether *sal1* growth phenotypes could be an output of deficiency or altered perception/signaling in growth-promoting hormones. For example, the *sal1* mutant rosette morphology resembles that of BR-insensitive or -deficient mutants (Caño-Delgado et al., 2004; Rodríguez et al., 2010) and *sal1* leaf venation patterning and lateral root formation shares similarities to mutants with altered auxin homeostasis (Robles et al., 2010). Indeed, BR-up-regulated genes are down-regulated in *sal1* and vice versa (Robles et al., 2010). Therefore, we supplemented soil-grown *sal1* mutant (*fry1*-) with the three growth promoting hormones – GA_3_, auxin [indole-3-acetic acid (IAA)] and *epi*-brassinolide (EBR) – individually and in combination (at least 10 biological replicates per treatment) from 2 weeks old onwards under 16 h photoperiod, as per method described (Ribeiro et al., 2012). We found that *sal1* rosette growth improved the most under the triple hormone treatment, resulting in the largest rosette area after 3 weeks of growth relative to the blank treatment control, followed by GA_3_ and EBR treatments (**Figure 1**). Importantly, the response to the hormonal treatments was different in *sal1*; with *sal1* growth complemented best by the triple hormone treatment compared to GA_3_ alone for wild type (seven biological replicates per treatment) (**Figure 1** and **Supplementary Figure S1**). Furthermore, none of the treatments tested here completely rescued *sal1* growth phenotypes.

Partial phenotypic reversion of *sal1* was also achieved by crossing the *sal1* mutant with the *allene oxide synthase* (*aos*) mutant, which has impaired JA biosynthesis and accumulation (Rodríguez et al., 2010). The resulting double mutants have rosette morphology that is partially reverted to wild type-like, specifically the rosette is less compact and has less anthocyanin accumulation but remain smaller than wild type. Hence, part of the altered rosette morphology of *sal1* is contributed by the over-accumulation of JA although Ishiga et al. (2017) reported impaired JA signaling in addition to lower levels of SA in *sal1* seedlings that correlate with increased its susceptibility to pathogenic attack.

When we analyzed two independent published microarray data of *sal1* in Col-0 ecotype background [*alx8*] (Wilson et al., 2009; Estavillo et al., 2011), we found at least 1500 genes commonly mis-regulated in the same direction (∼960 up-regulated, ∼550 down-regulated) despite some slight differences in plant age (juvenile-adult vs. adult-flowering stages) and photoperiod (12 vs. 16 h light) (**Supplementary Table S2**). GO enrichment analyses using DAVID Functional Annotation Clustering revealed that genes involved in translation, defense response, ADP binding and tryptophan biosynthetic processes are significantly enriched in *alx8* transcriptomes (**Supplementary Table S3**), which correlates with the pleiotropic *sal1* phenotypes discussed earlier. A substantial number of different hormonal biosynthetic and degradation/inactivation genes (as annotated in the AraCyc/Plant Cyc database) are mis-regulated in the *alx8* microarray data. These include up-regulation of genes involved in biosynthesis of ABA, auxin, JA, and GA (*CYP88A3* – *ent*-kaurenoic acid hydroxylase 1); while one BR-inactivation gene and a key chloroplast-localized GA biosynthetic gene *GA Requiring 2* (*GA2*; also known as *ent*-kaurene synthase) are down-regulated (**Supplementary Table S4**). Since the GA biosynthetic gene *CYP88A3* that is up-regulated in *alx8* is positioned downstream of GA2 in the GA biosynthetic pathway, the up-regulation of *CYP88A3* is likely a feedback response to the reduced substrate availability.

In order to verify if these transcriptomic alterations impacted the *sal1* hormonal profile, we performed quantifications of six different hormones (ABA, JA, auxin, GA, cytokinin, and SA) from leaves of two *sal1* mutant alleles (*alx8* and *fry1*-6) in Col-0 (*n* = 3 to 5 biological replicates) as per method described in (Kanno et al., 2010) (**Table 1**). Our data revealed for the first time that the *sal1* mutants have significantly higher auxin (IAA) and lower GA (GA_4_) levels relative to the Col-0 control, which correlates with the transcriptomic data. Additionally, we observed higher means and substantive increases in the standard deviations relative to mean for the stress hormones (JA-Ile, JA, and ABA), to an extent that renders JA and JA-Ile levels in *sal1* to be not statistically different compared to wild type. Variable increases in ABA and JA have already been reported (Rossel et al., 2006; Rodríguez et al., 2010; Ishiga et al., 2017; Pornsiriwong et al., 2017) and this variability would suggest indirect genotype/development/environmental influences on these hormonal content more than direct PAP signaling regulation of biosynthesis. In this context it is relevant that the stomatal regulation by PAP is primarily due to changes in ABA signaling, not ABA levels (Pornsiriwong et al., 2017). In contrast, there was no change in the content for SA and cytokinins (*t*-zeatin and isopentenyladenine) in *sal1* lines compared to that of wild type (**Table 1**). Collectively, our hormonal profiling data indicate that *sal1* has altered hormonal homeostasis not only in an abiotic stress hormone (ABA) but also in growth regulating hormones (auxin and GA) during the juvenile-to-adult stage of development, which are consistent with the *sal1* transcriptome (**Supplementary Table S4**) and growth complementation by GA and triple hormone treatment (**Figure 1**).

**Table 1:** Hormonal quantification results in fold-change for *sal1* mutants in comparison to Col-0 wild type at 4 weeks old.

**Hormones**	**Col-0**	***alx8***	***fryl-6***
Salicylic acid	1.06 ± 0.57	1.70 ± 0.57	1.41 ± 0.26
JA-isoleucine	0.99 ± 0.30	4.55 ± 5.17	3.96 ± 2.14
Jasmonic acid (JA)	0.96 ± 0.36	11.87 ± 10.36	7.98 ± 6.08
Indole acetic acid	1.05 ± 0.25	3.67 ± 0.91^∗∗^	5.03 ± 1.47^∗∗^
Abscisic acid	0.97 ± 0.08	1.88 ± 0.37^∗∗^	1.73 ± 0.43^∗∗^
Gibberellin (GA4)	0.95 ± 0.11	0.40 ± 0.07^∗∗^	0.48 ± 0.09^∗∗^
t-Zeatin	0.99 ± 0.16	0.92 ± 0.07	0.84 ± 0.20
2-Isopentanyladenine	0.96 ± 0.10	0.86 ± 0.29	0.83 ± 0.39

*Hormonal profiling was performed on rosette tissues as per methods detailed in Kanno et al. (2010). Means of fold-change ± standard deviation were reported. *n* = 3 to 5. Significant difference between mutant and wild type was tested based on ANOVA – Dunnett test, where ^∗∗^*p*-value < 0.01*.

When considering our hormonal quantification data together with published values it is likely that the effect of PAP accumulation on hormonal homeostasis could be developmental-stage dependent. In contrast to our IAA quantification at the adult stage, Chen and Xiong (2010) did not report differences in auxin levels between the *sal1* mutant and wild type at a more juvenile stage. However, *sal1* mutant has altered auxin perception (Robles et al., 2010), which could contribute to the increased IAA levels quantified at a later developmental stage, assuming factors like ecotype and growth conditions are negligible. Similarly, no significant changes in SA levels were detected in our experiments in contrast to lower SA levels reported by Ishiga et al. (2017) in *sal1* 2-week-old seedlings. Since quantification of JA precursor production revealed that the extent of metabolic rate alteration in *sal1* differs between developmental stages (Rodríguez et al., 2010) – similar to wild type early on but increase later on – the disparities between the hormonal levels quantified could be due to the different developmental stages of *sal1* characterization. Whether or not the increased JA levels at later developmental stages contribute to a readjustment in SA levels in *sal1* require further investigation. Therefore, a more systematic and comprehensive profiling of hormones and adenosines throughout *sal1* and wild type developmental stages is necessary.

## Hunting for the Mediators of Sal1-Pap, Light and Hormonal Regulation of Plant Growth

What are the possible intersection points between SAL1-PAP chloroplast signaling, hormonal and light signaling? The signaling network is likely to be complex and comprise several components which have been demonstrated to contribute to *sal1* phenotypes; and participate in both hormonal and light signaling. In our view, tempting candidates would include, but not be limited to, the aforementioned RNA metabolism, *PHYB*, *HY5*, as well as DELLA and BR signaling proteins.

Our observation of lower GA levels in *sal1* could suggest for more abundant DELLA proteins in *sal1* compared to wild type since GA promotes DELLA degradation. DELLA proteins can physically interact with key hormone-responsive transcription factors such as JA ZIM-domain family proteins (JAZs) (Hou et al., 2010) and brassinazole resistant 1 (BZR1) (Bai et al., 2012; Gallego-Bartolomé et al., 2012; Li et al., 2012) to promote JA signaling, which can feedback into promoting DELLA expression (Wild et al., 2012) and repress BR signaling (**Supplementary Figure S2**). Intriguingly, DELLA proteins also mediate light responses *via* their interactions with phytochrome-interacting factors (PIFs) proteins (de Lucas et al., 2008; Feng et al., 2008). Furthermore, constitutive higher DELLA accumulation as a result of low GA levels can activate ABA biosynthesis (Piskurewicz et al., 2008) and confer drought tolerance to *Arabidopsis* (Colebrook et al., 2014). These features correlate with multiple reported growth and hormonal-related phenotypes of *sal1* discussed above, and it is interesting to note that transcript levels of a few DELLA proteins are up-regulated in *alx8* (Estavillo et al., 2011). However, whether this transcriptional up-regulation genuinely reflects increased protein abundance is unclear and detailed work is required to mechanistically dissect how PAP and GA together with other hormonal signaling are interlinked.

It will also be critical to further unravel the interaction between PAP and the secondary messengers ROS and Ca^2+^. Both of these messengers are known to be key components of hormonal signaling. They are also involved in light signaling and can crosstalk with one another for cellular signaling (Mazars et al., 2010; Gilroy et al., 2014; Görlach et al., 2015). It is intriguing that PAP has contrasting effects on ROS depending on cell and tissue type; inducing a ROS burst in guard cells associated with the restoration of stomatal closure in ABA insensitive mutants (Pornsiriwong et al., 2017) while suppressing ROS levels in vascular tissue (Wilson et al., 2009). Similarly, increased Ca^2+^ levels in roots was reported in one of the mutant alleles of *sal1* (Zhang et al., 2011), but exogenous PAP treatment does not induce any significant changes in cytosolic Ca^2+^ transients in guard cells (Pornsiriwong et al., 2017). Intracellular ROS and Ca^2+^ homeostasis are known to regulate cell growth in leaves and germinating seed (Conn et al., 2011; Leymarie et al., 2012); though the exact mechanisms are still unclear. It will be interesting to explore whether and how PAP can contribute to these processes.

## Concluding Remarks

The numerous studies on *sal1* to date reveal that the SAL1-PAP chloroplast retrograde signaling pathway in *Arabidopsis* can intersect with light signaling and hormonal signaling pathway in affecting, if not regulating, plant growth and development. While chloroplast retrograde, light and hormonal signaling pathways have been studied independently, and to some extent in a two-way crosstalk manner in the past; the SAL1-PAP pathway may provide the platform to investigate crosstalk between the three pathways altogether in regulating plant growth and development in response to environmental changes (i.e., sun vs. shade) at the molecular level.

## Author Contributions

SYP performed the transcriptomic mining and hormonal treatment experiment. DY and EN performed the hormonal quantification of *sal1*. SYP, KXC, GME, and BJP led planning, analysis, and manuscript preparation.

## Conflict of Interest Statement

The authors declare that the research was conducted in the absence of any commercial or financial relationships that could be construed as a potential conflict of interest.
