# Supplementary material for: The Arabidopsis SAL1-PAP Pathway: A Case Study for Integrating Chloroplast Retrograde, Light and Hormonal Signaling in Modulating Plant Growth and Development?
Source: Front Plant Sci. 2018 Aug 8;9:1171. doi: 10.3389/fpls.2018.01171 (PMC6092573; doi:10.3389/fpls.2018.01171)
Supplement: Supplementary file 2 [file Data_Sheet_1.PDF]

Supplementary Table 1: Summary of published *sal1* mutant alleles.

sal1 mutant alleles		Ecotype Background	References
Abbreviation	Full name / Description		
hos2	*High expression of osmotically responsive genes 2*	C24	Lee *et al.*, 1999; *Plant Journal*<br>Xiong *et al.*, 2004; *Plant Journal*
fry1-1 to -3	*Fiery 1-1 to -3*	C24	Xiong *et al.*, 2001; *Genes & Development*
alx8	*Altered expression of* APX2 *8*	Col-0	Rossel et al., 2006; *Plant, Cell, Environ.*<br>Wilson *et al.*, 2009; *Plant Journal*<br>Estavillo *et al.*, 2011; *Plant Cell*
fry1-6	*Fiery 1-6*	Col-0	Kim and von Arnim, 2009; *Plant Journal*
ron1-1	*Rotunda 1-1*	Ler	Robles *et al.*, 2010; *Plant Physiology*
fou8	*Fatty acid oxygenation up-regulated 8*	Col-0	Rodriguez *et al.*, 2010; *Plant Physiology*
supo1	*Suppressor of* PIN1 *overexpression phenotype*	Col-0	Zhang *et al.*, 2011; *Developmental Cell*
fry1-4, *fry1-5*	*Fiery 1-4, Fiery 1-5*	Col-0	Gy *et al.*, 2007; *Plant Cell*
fry1-7	*Fiery 1-7*	Ws	Hirsch *et al.*, 2011; *PLoS ONE*
