# Supplementary material for: The Arabidopsis SAL1-PAP Pathway: A Case Study for Integrating Chloroplast Retrograde, Light and Hormonal Signaling in Modulating Plant Growth and Development?
Source: Front Plant Sci. 2018 Aug 8;9:1171. doi: 10.3389/fpls.2018.01171 (PMC6092573; doi:10.3389/fpls.2018.01171)
Supplement: Supplementary file 3 [file Data_Sheet_3.PDF]

Supplementary Table 3: GO enrichment analyses of commonly mis-regulated genes in *sal1*-8 (*alx8*) based on DAVID Functional Annotation Clustering (using default settings)

Annotation Cluster	Enrichment Score	Gene Ontology	Description	P-value	Benjamini
1	16.76	Molecular Function	Structural constituent of ribosome	2.30E-20	1.70E-17
		Biological Process	Translation	0.03	0.60
2	5.12	Biological Process	Defense response	8.70E-08	4.90E-05
		Molecular Function	ADP binding	1.30E-07	4.60E-05
		Biological Process	Signal transduction	4.10E-03	0.19
3	2.54	Biological Process	Tryptophan biosynthetic process	1.90E-06	5.40E-04
		Molecular Function	anthranilate synthase activity	5.30E-05	0.01
		Biological Process	Cellular amino acid biosynthetic process	3.60E-03	0.18
4	2.45	Biological Process	Metabolic process	3.70E-04	0.04
		Molecular Function	Guercetin 7-O-glucosyltransferase activity	3.80E-04	0.04
		Molecular Function	Guercetin 3-O-glucosyltransferase activity	4.80E-04	0.05
		Biological Process	Flavonoid glucuronidation	6.10E-04	0.06
		Molecular Function	Transferase activity, transferring hexosyl groups	8.70E-04	0.08
		Biological Process	Flavonoid biosynthetic process	1.80E-03	0.11
		Molecular Function	UDP-glucosyltransferase activity	2.00E-03	0.15
5	2.21	Biological Process	Negative regulation of nucleic acid-templated transcription	1.20E-03	0.09
		Biological Process	Regulation of defense response	7.00E-03	0.30
		Molecular Function	Transcription corepressor activity	0.01	0.41
		Biological Process	Regulation of jasmonic acid mediated signaling pathway	0.02	0.45
