# Supplementary material for: The Arabidopsis SAL1-PAP Pathway: A Case Study for Integrating Chloroplast Retrograde, Light and Hormonal Signaling in Modulating Plant Growth and Development?
Source: Front Plant Sci. 2018 Aug 8;9:1171. doi: 10.3389/fpls.2018.01171 (PMC6092573; doi:10.3389/fpls.2018.01171)
Supplement: Supplementary file 4 [file Data_Sheet_4.PDF]

Supplementary Table 4: ***sal1*****-8 (*****alx8*** **allele) microarray data mining of mis-regulated hormonal biosynthetic genes**

Hormonal Genes Category	Probe set ID	Locus Identifier	Annotation	Fold Change	
				Estavillo *et al.*, 2011	Wilson *et al.*, 2009
ABA biosynthesis	255857_at	AT1G67080	ABA4 (ABSCISIC ACID (ABA)-DEFICIENT 4); intramolecular oxidoreductase	3.86	10.36
Auxin biosynthesis	250738_at	AT5G05730	ASA1 (ANTHRANILATE SYNTHASE ALPHA SUBUNIT 1); anthranilate synthase	2.02	4.56
	251847_at	AT3G54640	TSA1 (TRYPTOPHAN SYNTHASE ALPHA CHAIN); tryptophan synthase	1.74	2.88
	252827_at	AT4G39950	CYP79B2 (cytochrome P450, family 79, subfamily B, polypeptide 2); oxygen binding	7.48	11.64
	264052_at	AT2G22330	CYP79B3 (cytochrome P450, family 79, subfamily B, polypeptide 3); oxygen binding	5.86	9.8
	253534_at	AT4G31500	CYP83B1 (CYTOCHROME P450 MONOOXYGENASE 83B1); oxygen binding	2.45	2.72
	252677_at	AT3G44320	NIT3 (NITRILASE 3)	2.42	2.46
BR inactivation	267614_at	AT2G26710	BAS1/CYP734A1 (PHYB ACTIVATION TAGGED SUPPRESSOR 1); oxygen binding / steroid hydroxylase	-10.75	-3.51
GA biosynthesis	262891_at	AT1G79460	GA2 (GA REQUIRING 2); ent-kaurene synthase	-2.03	-3.72
	264586_at	AT1G05160	CYP88A3 (ENT-KAURENOIC ACID HYDROXYLASE 1); oxygen binding	3.66	4.95
JA biosynthesis	252618_at	AT3G45140	LOX2 (LIPOXYGENASE 2)	1.96	2.47
	257641_s_at	AT3G25760; AT3G25770	[AT3G25760, AOC1 (ALLENE OXIDE CYCLASE 1)];[AT3G25770, AOC2 (ALLENE OXIDE CYCLASE 2)]	2.05	3.23
	265530_at	AT2G06050	OPR3 (OPDA-REDUCTASE 3); 12-oxophytodienoate reductase	3.27	6.21
	259518_at	AT1G20510	OPCL1 (OPC-8:0 COA LIGASE1); 4-coumarate-CoA ligase	1.89	3.43
