# Supplementary material for: The Arabidopsis SAL1-PAP Pathway: A Case Study for Integrating Chloroplast Retrograde, Light and Hormonal Signaling in Modulating Plant Growth and Development?
Source: Front Plant Sci. 2018 Aug 8;9:1171. doi: 10.3389/fpls.2018.01171 (PMC6092573; doi:10.3389/fpls.2018.01171)
Supplement: Supplementary file 5 [file Image_1.pdf]

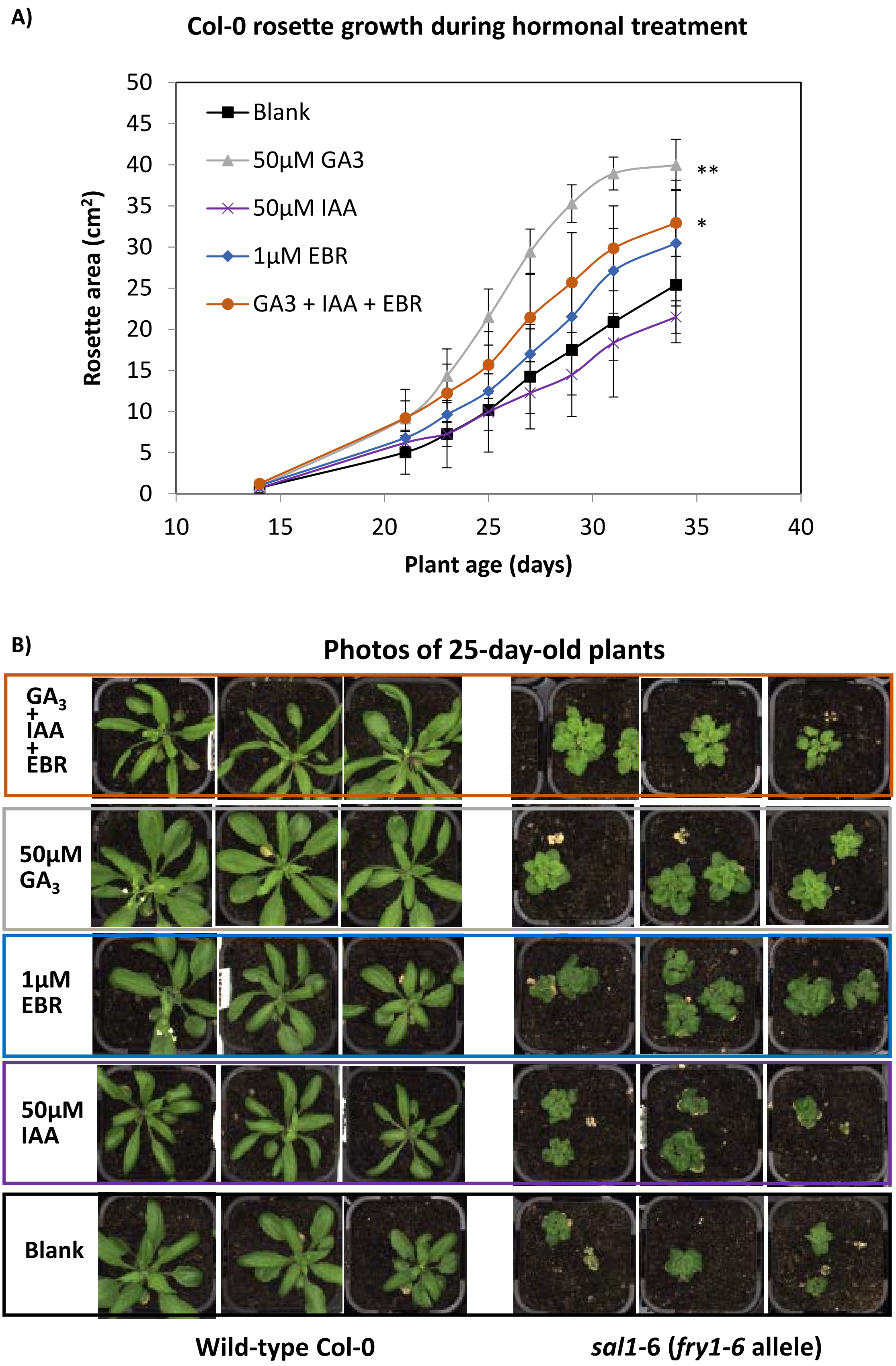

Supplementary Figure 1: Rosette growth under different hormonal treatment. A) Soil grown Col-0 were sprayed with different hormones [50 *µ*M of GA_3, 50 *µM* of IAA, or 1 *µ*M of EBR individually, or all of the three hormones together] or blank control [containing 0.5% (v/v) ethanol, which was used to dissolve the commercially available hormones, and 0.1% (v/v) Tween-20] from two weeks old onwards (three times per week). The rosette growth since treatment were quantified via LemnaTec Scanalyzer until bolting stage based on rosette area [full method see Phua et al. (2018)]. Average rosette area ± SD of seven plants per treatment are shown. Significant differences from blank treatment at 34 days old (ANOVA - Dunnett test [GraphPad InStat]) are indicated by ** for p<0.01 and * for p<0.05. **B)** Example photos of wild-type Col-0 and *sal1* plants at 25 days old (after 10 days of hormonal treatment).
