# Supplementary material for: The Arabidopsis SAL1-PAP Pathway: A Case Study for Integrating Chloroplast Retrograde, Light and Hormonal Signaling in Modulating Plant Growth and Development?
Source: Front Plant Sci. 2018 Aug 8;9:1171. doi: 10.3389/fpls.2018.01171 (PMC6092573; doi:10.3389/fpls.2018.01171)
Supplement: Supplementary file 6 [file Image_2.PDF]

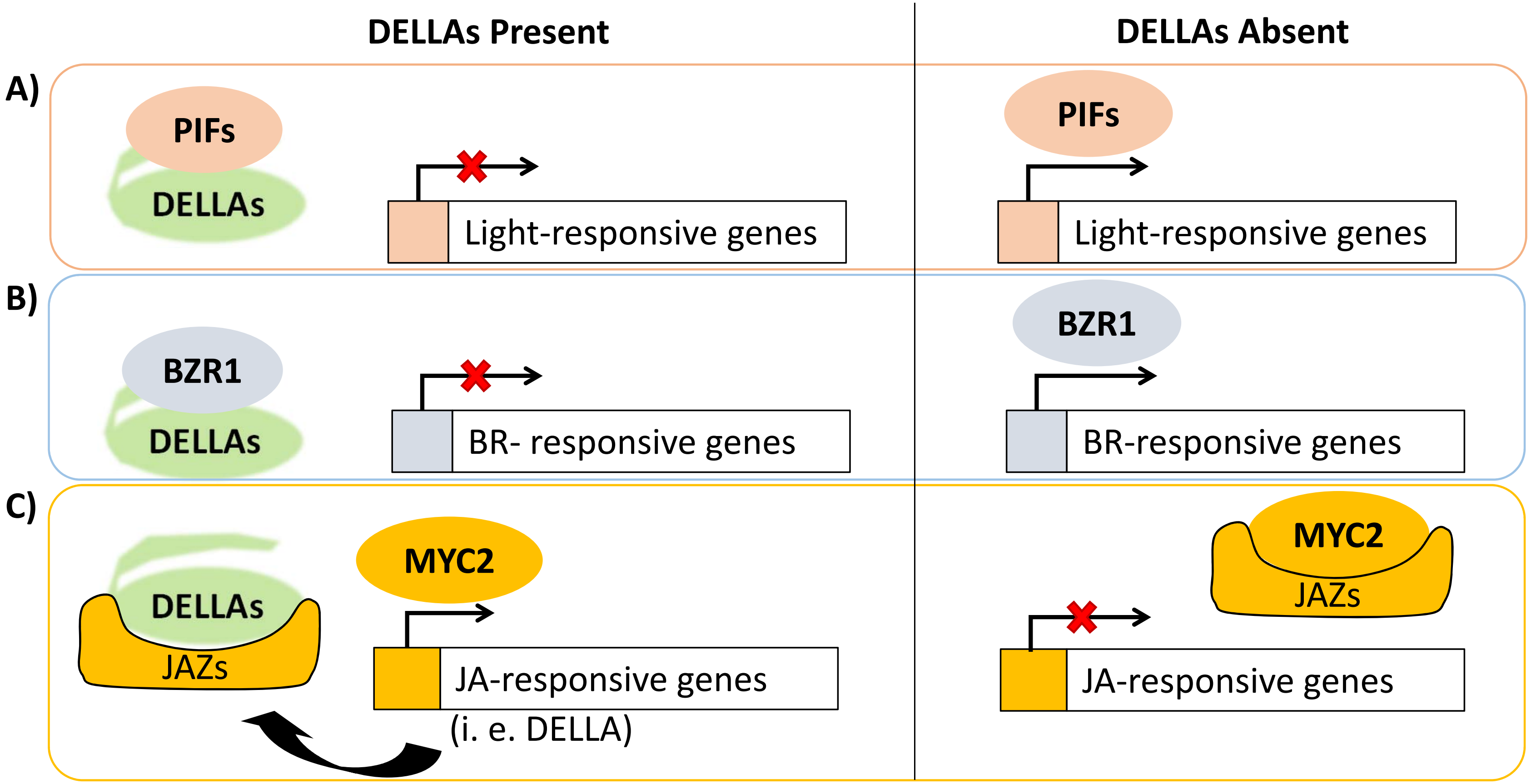

Supplementary Figure 2: DELLA proteins can physically interact with **A)** phytochrome-interacting factors (PIFs) to prevent them from binding to the promoter of target genes that regulate light-dependent hypocotyl elongation; **B)** brassinazole resistant 1 (BZR1) transcription factor that regulates the expression of brassinosteroid (BR)-responsive genes, preventing it from activating those genes; **C)** JA ZIM-domain family proteins (JAZs) and preventing it from inhibiting the activities of MYC domain transcription factor 2 (MYC2). As a result, jasmonic acid (JA)-responsive genes expression are promoted by MYC2, which includes some of the DELLAs, allowing a feed-forward regulation of JA-responsive genes. The presence of gibberellic acids (GAs) promotes the degradation of DELLAs and hence, reverses the inhibitory effects of DELLAs on PIFs, BZR1 and JAZs.
